# Facilitators and barriers to reach and enrollment into a medically tailored meals program within a section 1115 Medicaid pilot: clinic staff perspectives

**DOI:** 10.3389/fpubh.2025.1526564

**Published:** 2025-02-05

**Authors:** Sara C. Folta, Jessica Burch, Matthew Alcusky, Arlene S. Ash, Kurt Hager, Jean Terranova, Fang Fang Zhang, Oyedolapo Anyanwu, Zhongyu Li, Dariush Mozaffarian

**Affiliations:** ^1^Food is Medicine Institute, Friedman School of Nutrition Science and Policy, Tufts University, Boston, MA, United States; ^2^Community Servings, Boston, MA, United States; ^3^T.H. Chan School of Medicine, University of Massachusetts, Worcester, MA, United States

**Keywords:** food is medicine, medically tailored meals, enrollment analysis, qualitative research, health equity (MeSH)

## Abstract

**Introduction:**

Medically tailored meals (MTMs) are home-delivered, nutritionally tailored meals for individuals living with complex or advanced diet-sensitive medical conditions. In 2020, Massachusetts Medicaid implemented the Flexible Services Program (FSP) through a Section 1115 Demonstration, which funded novel nutrition programs, including MTMs, for high-risk patients through Accountable Care Organizations (ACOs). Little is known from the practitioners’ perspective regarding the facilitators and barriers to reaching and enrolling patients in MTM programs.

**Methods:**

We interviewed 19 staff across four ACOs that had implemented MTM interventions. Interviews were conducted from Feb to Aug 2023 and included staff who participated in patient screening, referral, or enrollment. The interview guide was informed by the Health Equity Implementation Framework. Interviews were recorded and transcribed and coded using NVivo software. We used directed qualitative content analyses. The study team identified and discussed common themes and presented them back to our ACO partners.

**Results:**

Staff described facilitators of and barriers to reach and enrollment related to several domains of the Health Equity Implementation Framework. For program (innovation) factors, facilitators included perceived positive effects on patient health outcomes and a relative advantage over both the status quo and other nutrition assistance programs; outreach by care team members rather than other staff; the eligibility criteria, which were viewed as appropriate and evidence-based; and the simplicity of the program, which aided communication with patients. Patient-related facilitators included patients being more in need of the program due to more severe illness and being more motivated to change dietary behaviors. Patient-related barriers included lacking a working phone or stable housing and concern about meals meeting taste and cultural food preferences. Staff-related barriers included limited time and especially knowledge about the MTM program.

**Discussion:**

This study highlights the perspectives of front-line staff during the implementation of an MTM program in a state-wide 1,115 Demonstration. Staff may require multiple trainings to gain full knowledge about the program and increase self-efficacy in describing it with sensitivity. These new findings elevate voices from front-line healthcare staff in MTM delivery and can help inform strategies for effective, equitable implementation of MTM programs.

## 1 Introduction

The integration of food and nutrition into healthcare delivery, sometimes called “Food is Medicine” (FIM), has shown promise in an increasing number of studies (1). Medically tailored meals (MTMs) are one type of FIM program that provides fully prepared, nutritionally tailored meals to individuals living with complex or advanced diet-sensitive medical conditions such as poorly controlled diabetes, heart failure, end-stage renal disease, HIV/AIDS, or cancer, and, often, social needs; the meals are home-delivered and designed by a registered dietitian nutritionist based on the medical diagnosis and a nutritional assessment (2). A growing body of research supports the effectiveness of MTMs in improving food security, diet quality, health outcomes (3–5), and in reducing healthcare utilization and costs (3, 6, 7). Given their impact, MTMs have the potential to address the World Health Organization’s Sustainable Development Goals (8), particularly Goal 2 Zero Hunger, since they are designed to increase access to healthful foods and can reduce food insecurity; and Goal 3 Good Health and Well-Being, by addressing non-communicable diseases. To date, however, MTM programs have gained greater traction in the United States (U.S.) than in other countries worldwide, and most studies have been conducted in the U.S. (9, 10).

Historically, MTM programs have been operated by community-based organizations and largely supported by grants and donations (1), making it challenging to scale MTMs within healthcare. More recently, in the U.S., several policy initiatives have piloted MTMs as a benefit within Medicaid, which serves individuals with low incomes and/or with disabilities, many of whom face food and nutrition insecurity. However, MTMs are not a covered benefit nationally in Medicaid [the recent policy initiatives are summarized in Mozaffarian et al. (9)]. In 2017, as part of its Section 1115 Demonstration, Massachusetts Medicaid and the Children’s Health Insurance Program (MassHealth) created Medicaid Accountable Care Organizations (ACOs), which bring together healthcare entities to provide coordinated care to Medicaid recipients while being held accountable for the cost and quality of care for a defined population of enrollees. In January 2020, MassHealth pioneered a $149 million, 3-year pilot initiative called the Flexible Services Program (FSP), which funds novel ACO-administered nutrition and housing programs, including MTMs. Similar Medicaid Section 1115 Demonstration waivers that include nutrition supports are being adopted by an increasing number of states, yet Massachusetts was the first U.S. state to cover MTMs within an 1,115 Demonstration.

Evidence from other nutrition programs indicates that even as policies aim to expand access to healthy foods, challenges can reduce uptake by eligible individuals. For example, the Special Supplemental Nutrition Program for Women, Infants, and Children (WIC) continues to enroll fewer households than are eligible (11). Data from participants and WIC providers has identified reasons for this discrepancy, including stigma, perceptions about the complexity of the application process, ineffective outreach methods, and failure to provide information about the program in potential participants’ native language (12–14). In WIC and other nutrition programs an understanding of such factors has led to program adjustments that meaningfully increased enrollment and participation (15–17). As access to MTMs expands, it is imperative to understand factors related to patient characteristics, clinic implementation, and those that are inherent to the program itself that may optimize or impede enrollment. Consideration of these factors from an equity perspective is also important to understand whether certain programmatic or structural factors may result in higher or lower enrollment among specific subgroups of the eligible population.

The front-line practitioners who screen, refer, and enroll patients into MTM programs have a unique perspective on the corresponding impacts, benefits, and facilitators and barriers to reaching and enrolling patients. To gain these insights, we conducted qualitative key informant interviews with staff responsible for screening, referring, or enrolling patients into MTM programs across several Massachusetts ACOs. The findings can help inform future FIM programming in other U.S. states and can provide considerations for other contexts as other countries begin to adopt these programs.

## 2 Methods

### 2.1 Setting, intervention, and sample

Of the 17 newly created ACOs that were invited by MassHealth to participate in the Flexible Services Program, 11 included a major focus on MTMs and partnered with Community Servings, a non-profit organization with over 30 years of experience in providing MTMs and a state-wide reach. MTM plans usually include lunch, dinner, and snacks for 5 days per week (10 meals per week), delivered to the home. The meals are designed by a registered dietitian nutritionist based on the patient’s disease diagnosis and a nutritional assessment. For enrolled patients in FSP, MTMs are generally provided for 6 months. To be eligible, members need to have both food insecurity and a qualifying medical condition, including but not limited to diabetes, cardiovascular disease, high-risk pregnancy, or behavioral health conditions.

We interviewed 19 staff associated with four of the 11 ACOs which focused heavily on MTM interventions within their Flexible Services programs. These four ACOs had agreed to partner with the research team on a larger, multimethod study of the impact of MTMs offered through FSP in Massachusetts. We interviewed both members of care teams, such as community health workers, and ACO administrators, such as Flexible Services program managers. All interviewed staff had a role in the referral, screening, or enrollment of patients. Interviews were conducted from February to August 2023; therefore, although the FSP began in 2020, these interviews describe implementation after the COVID-19 emergency had subsided. Patient interviews were also conducted and will be analyzed and reported separately.

### 2.2 Interview procedures

The semi-structured interview guide was informed by the Health Equity Implementation Framework (18, 19), which prioritizes assessing the impact of implementation on health equity within healthcare settings. Table 1 contains the Framework domains, related interview questions, and analysis codes with their definitions (it also contains a summary of findings, as described below, so that the table would provide a transparent trail of how the findings were derived). Questions were developed by the team’s qualitative expert (SCF) with input from the research team.

**Table 1:** Health Equity Implementation Framework domains, related interview questions, codes and definitions, and summary findings.

Health Equity Implementation Framework domain^a^	Interview questions	Codes and definitions	Summary findings
Program factors (characteristics of the innovation): observable results, relative advantage, process, clarity/simplicity	Talk about your impressions of the program overall. How effective do you believe the program has been for meeting the needs of patients? What do you think were the advantages of the medically tailored meals program, if any, over any similar programs for patients? What do you see as drawbacks of this program, if any, compared to any similar programs for patients? In your opinion, how does the medically tailored meals program compare to other Flexible Services programs that you offer? Describe any challenges in the screening and referral of patients. Which members of your team were most responsible for program-related duties? Describe how easy or hard it was for patients to understand the medically tailored meals program in terms of its purpose and logistics. How does the State’s eligibility criteria for the Flexible Services Program align with how your ACO would ideally determine a member’s need for medically tailored meals?	Ability to meet patient need: program’s ability to help patients Comparison to other programs: including other nutrition programs and other Flexible Services programs Screen and referral: barriers and facilitators, description of process Eligibility criteria: how the State’s eligibility criteria align with how the ACO would ideally determine a member’s need for MTMs	Staff had positive perceptions of the program, including its impact on patient health metrics and relative advantage over both the status quo and other nutrition programs Outreach is easier when a care team member is involved (vs cold-calling from a list of eligible patients) Program simplicity facilitated outreach Eligibility criteria viewed as appropriate
Patient factors: culturally relevant factors, demographics, socioeconomic status, beliefs and preferences	What factors might have impacted the ability to reach the patients who need this program the most? How might the program be changed to address these issues? Talk about whether you think there is any stigma related to participation in the program. What are common barriers that patients faced in participating in the program? In what ways, if any, did social determinants of health affect patients’ ability to participate in this program? [By social determinants of health, I mean the conditions where people are born, live, work, and play -- things like economic stability, access to quality education, their neighborhood and built environment, and their social and community context.] How might that be mitigated in future medically tailored meal programs? In general, how receptive were patients to the program? For patients who were less receptive, what were their concerns?	Patient factors affecting program reach: social, economic, or medical factors, food and cultural preferences, motivation, family context Improvements in program reach: ideas to increase program reach and support Patient receptiveness: description of how receptive patients were to the program	Patient factors such as being sicker or being more motivated to change facilitated enrollment Barriers to reach and enrollment included not having access to a working phone, having unstable housing, and taste and cultural preferences
Staff factors (provider factors): provider knowledge and attitudes, skillsets	What components of the program do you wish you knew more about, if any? What is required for [ACO/clinic] participation in the medically tailored meals program in terms of personnel? Approximately how much extra time was spent by staff members with each program participant, compared to how much time they would otherwise spend during a visit? Do you feel you need additional training to effectively screen and refer patients to the program? Did your clinic receive or provide any trainings for staff related to this or other programs to address health-related social needs of your members?	Staff knowledge: what staff members know (or do not know) about the MTM program Personnel and time: description of staffing requirements and responsibilities and time spent screening and referring patients Training: content and frequency of program-related trainings	Barriers to screening and enrollment included staff knowledge and time

^a^ Woodward et al. (19).

Interviews were conducted online via the Zoom videoconferencing application by the team’s qualitative expert and two others on the team who had training and experience in qualitative methods (ZL and OA). Interviews lasted an average of 50 min. All interviews were conducted in English. Participants were offered a $25 gift card for remuneration. The protocol was reviewed and deemed exempt by the Tufts University Health Sciences Institutional Review Board. Informed consent was obtained verbally from all participants.

### 2.3 Analysis

All interviews were recorded and transcribed, and then coded using NVivo software (version 12, QSR International, Doncaster, Australia). We used a directed qualitative content analysis approach, which is deductive (20). We drafted an initial codebook based on the interview guide. We then conducted a review of the transcripts and added codes for topics that arose in the data. Once the codebook was established, two coders independently coded one randomly selected transcript. We determined inter-rater reliability, with a kappa coefficient of 0.7 or greater at each code deemed as acceptable. Based on this testing, 84% of the codes met this threshold. A team review of the codes that failed to reach this threshold revealed minor differences in interpretation, informing revisions of the codebook by clarifying code definitions. The codebook remained stable after this point, reflecting code saturation (21). Two coders (SCF and OA) then independently coded three additional transcripts, randomly selected from each of the three remaining ACOs, so that all four ACOs were represented in the sample of double-coded transcripts. We again determined inter-rater reliability to ensure that the 0.7 threshold for Cohen’s kappa was reached. The remaining transcripts were coded by one study team member (OA). We examined the data for common themes, which were discussed among the study team and presented back to the ACO partners.

## 3 Results

### 3.1 Participants

All interviewed staff had a role in the referral, screening, or enrollment of patients per the eligibility criteria. Of the 19 staff, eight were ACO administrators, and 11 were clinical care team members (Table 2). Most (11 of the 19) had been in their position for 1–3 years, and six had been in their position for 4+ years. The mean age was 38.8 years. Sixteen of the 19 staff were female, and six (about one-third) were non-white.

**Table 2:** Clinic staff characteristics (*N* = 19).

ACO affiliation, *n*	
ACO 1	4
ACO 2	4
ACO 3	7
ACO 4	4
Staff type	
ACO Administrator	8
Care Team Member	11
Age in years, mean (min-max)	38.8 (25–63)
Gender female, *n*	16
Race, *n*	
Black	2
White	13
Asian	2
Other	2
Ethnicity	
Hispanic, *n*	1
Non-Hispanic	17
Don’t Know	1
Years in current position, *n*	
<1	2
1–3	11
4+	6

The staff described multiple factors related to the ability to fully reach and enroll patients who would benefit from the MTM program. Questions were based on the Health Equity Implementation Framework domains (18), and findings are organized by domain: characteristics of an innovation, which we refer to as program factors; patient factors; and provider factors, which we refer to as staff factors since not all interviewees were frontline healthcare providers. Table 1 provides the Framework domains and a summary of findings, as well as the related interview questions and analysis codes with their definitions to provide a transparent trail from the Framework domains to the findings.

### 3.2 Program factors

Characteristics of the innovation (the MTM program) include observable results, which we report as staff perceptions about the MTM program’s impact, as well as its relative advantage over the status quo and similar programs. These are characteristics that influence the adoption and implementation of an innovation (22) and, therefore, affect whether reach and enrollment will be effective. We also provide findings on the processes used for reach and enrollment and the program’s perceived clarity (simplicity) (18, 23), and staff perceptions on how these factors affect reach and enrollment.

#### 3.2.1 Perceptions of the program’s impact

Staff described perceptions of the program’s impact, including a positive impact on patient health metrics (i.e., there were observable results of the MTM program). They could readily recall examples of impact, and most used an enthusiastic tone, indicating a highly positive attitude toward the program.

*“Then also, just their health measures, reducing their A1C, reducing their weight, improving their blood pressure. I’ve noticed that from my conversations with the doctors and just looking at the data in the chart, it has improved their health.” Complex Care Manager, ACO 3, age 33.*

*“We get to see A1Cs decrease and see the actual effects of patients getting these services and how it can positively impact food security, health outcomes, et cetera… It’s one of the parts of my job that is really great because I get to see positive outcomes and we do not always get to see that in the healthcare world.” Social Work Manager, ACO 2, age 31.*

*“…we are actually seeing a reduction in their food insecurity or we are seeing a decrease in their food or health issues, we also do see a reduction in going back to the hospital or emergency units.” FSP Coordinator, ACO 4, age 25.*

Staff also expressed several perceived relative advantages of the MTM program over both the status quo and other nutrition programs like Meals on Wheels, grocery store gift cards, or food pantries, including the home delivery and the nutrition education component. Many described the relative advantages specifically for patients who lacked the ability to obtain or cook food for themselves – tailoring to this population was a key aspect.

*“I personally, just from a social work and clinical perspective, think it’s fantastic to be able to cut out some of the barriers that folks face when it comes to nutrition because of transportation. Like, the fact that it’s home delivered I think is amazing and that it’s tailored to them.” Social Worker, ACO 2, age 38.*

*“For patients who have mobility constraints, this program is better than say, a grocery store gift card, because it’s delivered to their home, they do not have to leave their house. We do find that transportation is a significant barrier for patients in going grocery shopping, so this does eliminate that barrier. Then for patients who do not know how to cook or do not have time to cook, or are unable to stand long enough to prepare their own food, this program is advantageous to something like a produce box because the meal is already prepared for them.” FSP Coordinator, ACO 3, age 27.*

*“For other programs that are similar, home-delivered, the only one that I can really think of would be something like Meals on Wheels for our seniors. I have that option, but like I said, I do not feel like it gives that same focus on the medical and they do not get the nutrition education.” Social Worker, ACO 2, age 37.*

#### 3.2.2 Program factors influencing reach and enrollment

In terms of process, each ACO had multiple pathways for referral and enrollment. In some cases, the ACO would generate a list of eligible patients from their database, which was then given either to an ACO staff member to contact patients or to a care team member for outreach. In other cases, clinic staff in a coordinated care team would identify patients during visits and refer them to ACO staff for screening. ACO staff described outreach as generally easier when a care team was involved. Their descriptions of each method suggest that the existing relationships, rather than any logistical factors, were the basis for the difference between the list method (“random person calling”) and the referral method (“warm handoff”).

*“Yes, I would say that, because we are engaging members in the care management program and not doing cold calls or working from an outreach list, we have had high success with connecting members to the services since there’s already a care team that is engaging the member, they are engaged with their community health worker and their nurse care manager on the care management program. Then when the community health worker is talking to the member about their social needs, they are able to then make a referral to Community Servings, and then from there it’s a warm handoff.” FSP Manager, ACO 4, age 28.*

*“I think we do have a list of patients who are technically eligible, but maybe they are not so connected with [ACO], and our team calls some of those patients. Then, just being able to reach them is like-- you cannot always reach them because we are like a random person calling.” Population Health Program Manager, ACO 3, age 33.*

Staff across all four ACOs perceived the program’s eligibility criteria as appropriately focused on higher-need patients and on the scientific evidence for prioritizing patients who would realize the greatest benefit. Their positive attitudes implied that the eligibility criteria facilitated the outreach conversations.

*“Everybody enrolled has to have a complex physical health condition that is diet related and that has been shown through research to be benefited by a meals program, because Community Servings has a lot of research on this topic. Food is medicine, meal delivery has been researched for at least a decade, at least, I’d say. Our list of complex health conditions are those that would benefit directly from a healthy diet.” FSP Coordinator, ACO 3, age 27.*

Staff said that the program’s simplicity and the materials’ translation into multiple languages also facilitated outreach conversations.

*“They needed some clarity when it came down to certain information [like how many days per week and what type of meals] but once they received that clarity they were able to understand it pretty well.” Community Health Worker, ACO 1, age 31.*

*“I think it’s pretty easy for patients to understand. I do spend a little bit more time explaining it to patients whose language is not English, but I think that’s just part of the process. I think Community Servings also has all the materials translated so they can communicate with these patients effectively as well.” FSP Coordinator, ACO 3, age 27.*

A key program factor is that there is no cost to patients for the meals. However, staff said they had to work to overcome a misperception that the program had a cost associated with it:

*“In the beginning, some patients when you discuss it with them, they always think that there’s some fee involved, that they are not going to get it for free so you have to be very clear in explaining this is a free program.” Complex Care Manager, ACO 3, age 51.*

*“We are very brief and concise as much as possible to make sure the patient understands the type of program that it is and just letting them know it is free. That is one of the things that they do ask.” Community Health Worker, ACO 1, age 31.*

### 3.3 Patient factors

Staff described patient factors that facilitated enrollment, including having more severe disease and being highly motivated to change their dietary patterns but lacking the resources to do so. Their descriptions suggest that it was a certain type of patient, not all eligible patients, in whom these facilitating factors were present.

*“… I think the people who are also a little bit sicker are more willing, especially if they have cancer or something else and they just cannot cook and they do not have someone to cook for them, I think they are also more willing to accept the meals.” Community Care Manager, ACO 3, age 42.*

*“Again, I think it really goes back to this motivation piece because there is some behavior change involved here... If somebody is really motivated to make a change, I think they are 1,000% on board and are completely engaged with us. Like I said, sometimes people feel motivated to engage because they are trying to manage food insecurity because they just do not have enough money to pay for their food throughout the month. This is a program that supplements their food in that way. They’re motivated to engage because of that.” Complex Care Manager, ACO 3, age 37.*

Staff also described multiple patient factors hindering the program’s ability to reach and enroll patients who most needed it. Although these factors were related to individual patients, they were situated within the outer level of the Health Equity Implementation Framework, which encompasses societal influences (18), and were linked with social determinants of health. Many staff members used language that indicated that they viewed these patient factors within a broader context, with some staff specifically referring to social determinants. As an example of this type of factor, not having access to a working telephone was described as a major barrier to reaching and enrolling eligible patients.

*“I think again, social determinants barriers, a lot of my patients are living with low income and when their phone gets shut off because they have not been able to pay the bill, that creates a barrier…” Complex Care Manager, ACO 3, age 37.*

*“… Or we have a lot of members who might change their phone number or run out of minutes on their cell phone, so they might be transient and so difficult sometimes to be able to connect with them.” Community Health Worker, ACO 1, age 51.*

*“… But by and large, the members that we are working with live really complicated lives. What sounded good today, maybe by Wednesday or Thursday when the Community Servings person’s calling, their phone has already been disconnected”. Community Health Worker, ACO 4, age 51.*

A few staff said they addressed this by following up during in-person clinic visits.

*“I think that we, in addition to the multiple attempts of outreach, knowing that somebody may be coming in for an appointment, there’s been a time where I saw someone was scheduled to be coming in, so I can try to reach them at that time. I think just being creative of knowing, trying to catch people when we have access to them is helpful.” Social Worker, ACO 2, age 37.*

Unstable housing was another factor that impacted reach and enrollment since it made the patients harder to communicate with about the program and because the program requires a valid address to deliver the meals. Staff could refer patients to another FSP that assists with housing, although they said it was a long process that did not always result in a positive outcome. The MTM program also requires that a patient has access to adequate cold storage and heating facilities, which was not always the case.

*“I remember one patient, I think it was she could not afford a microwave so some of the meals options, she could not receive because of that and then another patient lived in like a single room occupancy, so she had her own room, but she had to share a kitchen with many other people. Then there wasn’t fridge space that allowed her to have the frozen or I guess there wasn’t freezer space for her to have frozen meals and then enough fridge space for some of the other meals. I guess that was a barrier.” Complex Care Manager, ACO 3, age 33.*

*“I actually do have a patient that I wanted to enroll her. She has many medically complex needs, and she has food insecurities, and I wanted to enroll her in that... She’s staying with a friend and they are not supposed to have anybody else living there, so she will not give me her address. We tried to do a workaround with that and if she had family, somebody in the area that we could use another address that we could have the food delivered, but she did not have anybody else in the area... If they do not have permanent housing, that is definitely a drawback and a deterrent to them getting it.” Complex Care Manager, ACO 3, age 51.*

*“There are oftentimes, actually, I’ll call out a member that I’ve been working on this week who lives in their car. This member has very complex physical health conditions, and I would say would be the perfect candidate for medically tailored meals, but really because they live in their car. They sometimes are able to stay with families and friends and they are couch surfing, but again, just a really transient situation. They’re not able- they do not have a stable address where they can receive the meal deliveries, so I would call that out.” FSP Manager, ACO 4, age 28.*

Based on their experiences, staff suggested several strategies to address these issues, such as delivering the meals to a shelter or other community location, delivering fewer meals at a time, or providing meals that do not require refrigeration.

*“I do not know if they have a program where they could deliver to homeless shelters or a certain site, or just deliver one meal or couple meals once a week instead of the all 10 for people who do not have the refrigeration for it… I wonder if they have, not shelf-stable meals, but meals or food that does not need to be refrigerated… Even a place they could pick it up every day, a meal site. I know there’s some other meal sites in the area.” Community Care Manager, ACO 3, age 42.*

Another barrier to enrollment that staff described was patients’ taste preferences, including cultural preferences, which made them less likely to want to enroll.

*“When I first describe a meal, even though they are experiencing food insecurity, they are not super excited [chuckles] about it because they are like, ‘Ugh, I’m not going to like the meals.’ So, I describe, ‘Oh, no. No. No. It tastes better. It’s made from scratch.’ I do that with everyone.” Population Health Program Manager, ACO 3, age 33.*

*“…More so just like, ‘I do not need that. I prepare my own food. I like my food a really specific way.’ We hear that one a lot. I think patients who eat in a specific way is probably the biggest one. They’re very particular about how they like food, so that’s an instant deterrent for them. Those are probably the biggest ones.” Social Work Manager, ACO 2, age 31.*

*“We had some patients recently who follow a Halal diet and had difficulty figuring out what meal plan would work for them because they eat Halal foods and Community Servings does not cater to that diet plan.” FSP Coordinator, ACO 3, age 27.*

*“We serve a very diverse population, and the question that we always get from members is, ‘Oh, what are these meals?’ Will the members like them? Are these familiar ingredients, familiar spices, familiar tastes to all the different ethnic cultures that we serve?” Community Health Worker, ACO 1, age 25.*

Staff described additional challenges that indicated a need to communicate clearly and sensitively during the screening and referral process, including patients being confused by the food insecurity screening questions or answering them inaccurately because of concern about being stigmatized, and taking offense at the offer of healthy food support. Their description of these challenges and their tone in describing them suggested that while staff attempted to address these factors, they did not feel fully comfortable overall at doing so.

*“There would be some members who they’d be completely eligible and they’d benefit from the meal, but they might not accept the meal because they do not want to accept anything. There are some like that just do not want. They do not feel they need it, more along the lines of trying to be independent.” Community Health Worker, ACO 1, age 51.*

*“Some patients from other cultures, I have to repeat the question [about food insecurity] because the first question is about worrying whether your food will run out. Some people do not really understand, like, ‘Well, no, it does not run out,’ because they go to the food pantry. It’s like, ‘Okay, but you did not have the ability to buy more food when you needed to, so you do screen positive for insecurity.’” FSP Coordinator, ACO 3, age 27.*

*“There’s also been some cultural concerns as well. Families that do get somewhat offended at the implication that their foods that they are preparing for their families aren’t healthy enough and that we are trying to come in and tell them that, ‘You should not be eating what you are cooking, or you should be eating what we are providing you as well.’” Complex Care Manager, ACO 3, age 33.*

### 3.4 Staff factors

The main staff factor that impacted reach and enrollment was knowledge, either about the eligibility criteria or the program itself. Some interviewees thought that some of the staff tasked with screening and referring may not readily recognize what constitutes a qualifying medical condition, which would hinder identifying (and therefore reaching) eligible patients.

*“I think just one of the things is we work with a lot of community health workers and stuff. They sometimes see this program as being more medicalized because they have to have a certain eligible health condition and they are not as comfortable identifying an eligible health condition because it is very specific. It’s easy to look in an electronic health record and see diabetes, but some of these other health conditions are-- if you have not gone to medical school, you are maybe not as comfortable identifying an eligible health condition. That has been a barrier to referring patients to the program.” Population Health Program Manager, ACO 3, age 33.*

A potential barrier to enrollment was staff knowledge about the meal options. Many staff said that they wished they knew more about the different meal options that Community Servings offers, particularly for patients with special dietary needs or cultural meal preferences. They said they often did not feel like they knew enough about the meals to address patient questions with confidence. Their main approach in these cases was to assure patients that someone from Community Servings would give them more details and answer their questions.

*“Yes, I’ve seen the meal-- I do not know about all of their menus. I know it’s nutritionally based and it’s locally sourced, but I do not know how the diabetic menu differs from the renal menu and things like that.” Community Care Manager, ACO 3, age 42.*

*“You just rely that someone else explain a little bit further. It probably should not be like that. I think that, from the patient perspective, they were definitely open and interested, but I wasn’t able to provide much on the spot about details of the program.” Community Health Worker, ACO 1, age 25.*

*“I serve large population of immigrants, like Cape Verdeans, Haitians, Hispanic, Portuguese, and a lot of them I do not know if the food meets their culture. Do you know what I mean? That, I do not know.” Vice President for Policy, ACO 4, age 57.*

Many staff said they had received an initial one-time training and felt that additional training would be useful. Their language and demeanor suggested a positive attitude toward the possibility of this additional training. They also suggested that reach and enrollment could potentially be improved by training other healthcare providers such as primary care physicians and nurses who work directly with patients.

*“I wonder if we were to tour Community Servings or if we were to have somebody come talk to our team so that we could get some of those nuanced answers to some of the questions that patients might ask that we may not know or even to just see and taste the meals being able to have more tools to sell it kind of a thing… Just having more people know about it. If the nurse knows, the medical assistant knows, the doctor knows, and the patient said something to one of those people, then there’s the connection. If they do not know about it, food insecurity might come up, but they do not have that to offer. We might miss that person in that opportunity. I think for us not just being siloed to just the social work staff managing and making sure that the rest of our teams know that for our ACO patients, there are alternatives for food insecurity.” Social Worker, ACO 2, age 38.*

*“…maybe like a refresher every year being like, ‘Remember this program exists.’ After six months or so. It’s not often the highest thing on my radar.” Community Care Manager, ACO 3, age 42.*

Limited staff time was also described as a barrier to full outreach, although less so than challenges around knowledge described above.

*“I have a much larger team than I did in the earlier days of medically tailored meals, but they are still tasked with a bunch of other things every day… I would love for every patient who is eligible to be able to receive services, but we can only reach out to so many people at once.” Social Work Manager, ACO 2, age 31.*

*“Then I think for me to fill it out, it does not take long at all, and then to fax it over, I’d say. But if you have 300 people you need to reach out to, those times can add up really quickly and you do not know if it’s going to be someone that you are going to have a 10-min conversation with or 20 min conversation with. It’s a little unpredictable. I think it’s a personal thing where people put it in their workflow and areas that work for them. Because it’s not part of our just general workflow, I think that’s the piece that’s hard for us, because we have our general workflow and then we have, oh, yes, medically tailored meals and oh, yes, casa project or the housing service.” Social Worker, ACO 2, age 38.*

## 4 Discussion

In this investigation of front-line staff implementing screening, referral, and enrollment into a major state Medicaid 1,115 Demonstration project, staff expressed highly positive perceptions of the MTM program. They also described several factors that were hindering the ability to fully reach and enroll patients who were likely to benefit. These included patients’ lack of access to a working phone or stable housing, as well as concerns about taste and meeting cultural food preferences. Staff time and especially knowledge about the MTM program were also factors that hindered reach and enrollment. Findings suggest several modifications that might improve program participation and especially equitable access.

One of the most important facilitators was staff perceptions of positive health impacts for patients. This is consistent with the design of FSP and the MTM program: members not only had food insecurity but also a qualifying health need that the MTM program aimed to treat. These perceptions are consistent with the scientific evidence, which shows associations between MTM programs and reduced healthcare utilization (3). This is also consistent with staff’s positive perceptions around the eligibility criteria, which focus on the complex chronic conditions more likely to benefit from good nutrition. These features of MTMs were also perceived as unique and strong relative advantages over other nutrition programs, especially for patients who cannot easily obtain and prepare food.

Another identified facilitator of reach and enrollment was the MTM program’s simplicity, which made it easy for staff to describe it to patients and for patients to understand it during the referral process. Our results support the relevance of training staff responsible for outreach and enrollment to be able to describe the simplicity of the MTM program. Also, any future MTM program adjustments should consider how to retain simplicity since the perceived complexity of an innovation can hinder its adoption (22). For example, while further taste and cultural tailoring of the meals could help increase patient enrollment by increasing the program’s appeal, doing so could also add complexity to the process of enrolling patients and selecting meals. It may be important to consider measures to mitigate any added complexity.

The involvement of the care team in initial screening was viewed as a facilitator of enrollment, providing more personalization in outreach and engagement. Our results raise the possibility that an outreach model whereby members of a coordinated care team have the initial conversation with patients about the program may be more effective than electronic database screening followed by contact from a staff member outside of the care team. This could be formally evaluated in future studies to guide optimal outreach approaches. Our findings also suggest the value of increasing the awareness around FIM programs among multiple healthcare providers, further supporting calls for greater medical nutrition education so that physicians and other providers can effectively identify and refer patients (9). Since staff time was also described as a barrier, our findings suggest the importance of efficiently integrating screening and referral into the clinical workflow. Other states implementing Flexible Services programs through Medicaid 1,115 waivers may want to consider offering up-front administrative support to help with this integration. Administrative support may also improve the program’s overall uptake. One staff member recounted the poor uptake of the FSP in the first year. Although the initial year coincided with the onset of the COVID-19 pandemic, poor uptake was also attributed to challenges in administering the program, causing MassHealth to seek approval from the federal Centers for Medicare & Medicaid Services for authority to offer administrative support.

Our findings underscore multiple patient barriers to enrollment in MTM programs, suggesting adaptations to increase accessibility. From an equity perspective, the program may not fully reach patients with the greatest socioeconomic challenges, as indicated by the absence of a reliable phone or stable housing. Staff suggested several ways to address this issue, such as offering alternative drop-off locations, which could be incorporated proactively into future programs. In Massachusetts, housing support is also offered through FSP, although staff noted that it takes longer to put stable housing in place compared to food-related programs. Our results also support the need to address patient concerns around taste and cultural salience and for further staff training to ensure clear and sensitive communication, especially as related to food insecurity screening and stigma from participation in MTM programs.

It is important to consider how our results may or may not apply in other contexts outside of the U.S., given differences in socioeconomics, population characteristics, health and social systems, and the policy environment. We know of only one study outside of the U.S.: in 2023, Law et al. (24) published a protocol paper for an MTM study to be conducted in Australia. The patient factors that impact reach and enrollment, such as unstable housing, may be exacerbated in other contexts. For example, while housing insecurity is an issue globally, it is exacerbated in many low-to-middle-income countries by a lack of social safety net programs and limited housing stock (25). Our findings also suggest that as these programs are adopted in other countries, it will be important to conduct formative work with both patients and clinic staff to understand context-specific factors that may impact reach and enrollment. For example, no country is fully homogeneous regarding cultural diets and taste preferences, and in other contexts, differences may be more pronounced if additional factors such as religious diets play a larger role in the acceptability of, and therefore enrollment in, MTM programs. Finally, formative work with clinic staff will help assess the context-specific factors that may facilitate or impede outreach, such as the usual duties, workload, and time available for outreach.

This study had several strengths. It is one of few studies to qualitatively examine staff perceptions of an MTM program (26–28), and the first to our knowledge to do so in the context of a Medicaid Section 1115 Demonstration waiver. Staff were interviewed across four ACOs, increasing coverage and generalizability. Our semi-structured interview guide was informed by the Health Equity Implementation Framework, and we assessed program, patient, and staff facilitators and barriers. We achieved code saturation (21), suggesting the robustness of themes among the voices included in our sample.

Potential limitations should be considered. As with any qualitative study, the life experiences and assumptions of the researchers may have influenced the interpretation of the data. This was mitigated by reporting findings back to our ACO partners for their perspective and input. Some of the experiences and perceptions in Massachusetts may differ from those in other states, although many of the identified themes seem more universal. While the MTM program provided by Community Servings may be similar to other MTM nonprofits given their common accreditation standards (2), it may differ substantially from those provided, often by mail, from larger for-profit vendors of MTMs.

This study highlights the perspectives of front-line staff and “lessons learned” during the implementation of an MTM program in a state-wide Medicaid Section 1115 Demonstration. Our findings elucidate multiple factors related to the program, patient, and staff that must be thoughtfully considered to optimally reach and enroll eligible patients, guiding future strategies for program adaptation to increase reach and equitable access and suggest several policy and programmatic considerations for future programs. In future MTM-related policies, it will be important to consider including administration resources for healthcare organizations to be able to feasibly integrate outreach and screening into clinical workflows. It will also be important to ensure an adequate level of staff training; a one-time training is unlikely to be sufficient. Clear and evidence-based eligibility criteria will help facilitate the outreach and screening process. Relatedly, uptake of the program will likely be facilitated by appropriate targeting of patients who are food insecure and who have difficulty in obtaining and preparing food. Other patients may be more receptive to other types of FIM programs, such as produce prescription programs, which provide more choice. In designing future programs, formative research will help determine whether alternative meal delivery sites are necessary. It may also be necessary to provide patients with equipment (freezers or microwaves) to be able to participate. Formative research will also help ensure that outreach communications use appropriate channels, including alternative channels for patients who may not have access to standard ones (like a working phone), and that materials describe the program’s simplicity and are available in the appropriate languages. Findings are particularly relevant to other U.S. states and countries that are beginning to plan, launch, and implement MTM services.

## Data Availability

The datasets presented in this article are not readily available because of the specificity of the sample in terms of roles and geographic region and the potential for identification of study participants, even with names and other identifiers removed. Requests to access the datasets should be directed to Sara Folta, sara.folta@tufts.edu.
